# Supplementary material for: EnzML: multi-label prediction of enzyme classes using InterPro signatures
Source: BMC Bioinformatics. 2012 Apr 25;13:61. doi: 10.1186/1471-2105-13-61 (PMC3483700; doi:10.1186/1471-2105-13-61)
Supplement: Addtional file 5 — The Java code to format the data files, evaluate and predict. The file enzml_java_code.tar.gz contains the Java code used to format database data to ARFF and XML formats, to execute cross and train-test (jackknife) evaluations and to record evaluation results to database. More information is included in the readme.txt file and the Javadoc files. The code can be used with a MySQL database. To use a different database software, other JDBC drivers might be required. [file 1471-2105-13-61-S5.gz › java_code/enzml2011/doc/uk/ac/ed/inf/taweka/weka/DataSetDbLoader.html]

DataSetDbLoader


---


|  |  |  |
| --- | --- | --- |
| **Overview** \| **Package** \| **Class** \| **Use** \| **Tree** \| **Deprecated** \| **Index** \| **Help** | |  |
| **PREV CLASS**   **NEXT CLASS** | **FRAMES**    **NO FRAMES**     **All Classes** |  |
| SUMMARY: NESTED \| FIELD \| CONSTR \| METHOD | DETAIL: FIELD \| CONSTR \| METHOD |  |


---


## uk.ac.ed.inf.taweka.weka Class DataSetDbLoader

```
java.lang.Object
  uk.ac.ed.inf.taweka.weka.DataSetDbLoader
```

**Direct Known Subclasses:**: MulanDataSetDbLoader

---

``` public class DataSetDbLoader extends java.lang.Object ```

Loads data (instances, attribute values, class values) from database using
the queries in the data Properties.

**Version:**
:   28 Apr 2010

**Author:**
:   Luna De Ferrari luna.deferrari-at-ed.ac.uk

---

**Constructor Summary**

`DataSetDbLoader(DataSetManager manager)`


| **Method Summary** | |
| --- | --- |
| `void` | `createAndloadDataSet()`             Create a data set and load it with data from database |
| `java.util.Properties` | `getDataProperties()` |
| `DataSetManager` | `getDataSetManager()` |
| `uk.ac.ed.inf.utils.OneToManyMap` | `getInstanceAttributeData()` |
| `uk.ac.ed.inf.utils.OneToManyMap` | `getInstanceClassData()` |
| `uk.ac.ed.inf.utils.OneToManyMap` | `getMap(java.lang.String queryProperty)` |

**Methods inherited from class java.lang.Object**

`equals, getClass, hashCode, notify, notifyAll, toString, wait, wait, wait`

**Constructor Detail**

### DataSetDbLoader

```
public DataSetDbLoader(DataSetManager manager)
```


**Method Detail**

### createAndloadDataSet

```
public void createAndloadDataSet()
```

:   Create a data set and load it with data from database

---


### getDataProperties

```
public java.util.Properties getDataProperties()
```

---


### getDataSetManager

```
public DataSetManager getDataSetManager()
```

---


### getInstanceAttributeData

```
public uk.ac.ed.inf.utils.OneToManyMap getInstanceAttributeData()
```

---


### getInstanceClassData

```
public uk.ac.ed.inf.utils.OneToManyMap getInstanceClassData()
```

---


### getMap

```
public uk.ac.ed.inf.utils.OneToManyMap getMap(java.lang.String queryProperty)
```


---


|  |  |  |
| --- | --- | --- |
| **Overview** \| **Package** \| **Class** \| **Use** \| **Tree** \| **Deprecated** \| **Index** \| **Help** | |  |
| **PREV CLASS**   **NEXT CLASS** | **FRAMES**    **NO FRAMES**     **All Classes** |  |
| SUMMARY: NESTED \| FIELD \| CONSTR \| METHOD | DETAIL: FIELD \| CONSTR \| METHOD |  |


---
